# The association between maternal-child physical activity levels at the transition to formal schooling: cross-sectional and prospective data from the Southampton Women’s Survey

**DOI:** 10.1186/s12966-019-0782-9

**Published:** 2019-02-20

**Authors:** Kathryn R. Hesketh, Soren Brage, Cyrus Cooper, Keith M. Godfrey, Nicholas C. Harvey, Hazel M. Inskip, Sian M. Robinson, Esther M. F. Van Sluijs

**Affiliations:** 1 CEDAR and MRC Epidemiology Unit, University of Cambridge, Cambridge, UK; 2 MRC Lifecourse Epidemiology Unit, University of Southampton, Southampton General Hospital, Southampton, UK; 3 NIHR Southampton Biomedical Research Centre, University of Southampton and University Hospital Southampton NHS Foundation Trust, Southampton, UK

**Keywords:** Physical activity, Sedentary, Prospective, Children, Mothers

## Abstract

**Background:**

Physical activity decreases through childhood, adolescence and into adulthood: parents of young children are particularly inactive, potentially negatively impacting their children’s activity levels. This study aimed to determine the association between objectively measured maternal and 6-year-old children’s physical activity; explore how this association differed by demographic and temporal factors; and identify change during the transition to school (from age 4–6).

**Methods:**

Data were from the UK Southampton Women’s Survey. Physical activity of 530 6-year-olds and their mothers was measured concurrently using accelerometry for ≤7 days. Cross-sectionally, two-level mixed-effects linear regression was used to model the association between maternal-child daily activity behaviour at age 6 [minutes sedentary (SED); in moderate-to-vigorous physical activity (MVPA)]. Interactions with demographic factors and time of the week were tested; how the association differed across the day was also explored. Change in the association between maternal-child physical activity (from age 4–6) was assessed in a subset (*n* = 170) [outcomes: SED, MVPA and light physical activity (LPA)].

**Results:**

Mother-child daily activity levels were positively associated (SED: β = 0.23 [0.20, 0.26] minutes/day; MVPA: 0.53 [0.43, 0.64] minutes/day). The association was stronger at weekends (vs. weekdays) (interaction term: SED: β_i_ = 0.07 [0.02, 0.12]; MVPA: 0.44 [0.24, 0.64]). For SED, the association was stronger for those children with older siblings (vs. none); for MVPA, a stronger association was observed for those who had both younger and older siblings (vs. none) and a weaker relationship existed in spring compared to winter. Longitudinally, the association between mother-child activity levels did not change for SED and LPA. At age 6 (vs. age 4) the association between mother-child MVPA was weaker across the whole day (β_i_: − 0.16 [− 0.31, − 0.01]), but remained similar at both ages between 3 and 11 pm.

**Conclusions:**

More active mothers have more active 6-year-olds; this association was similar for boys and girls but differed by time of week, season and by age of siblings at home. Longitudinally, the association weakened for MVPA between 4 and 6 years, likely reflecting the differing activities children engage in during school hours and increased independence. Family-based physical activity remains an important element of children’s activity behaviour regardless of age. This could be exploited in interventions to increase physical activity within families.

## Background

Physical activity plays an important role in health and prevention of disease, with active adults and children tending to have decreased adiposity, more favourable cardiovascular risk profiles and better psychological outcomes than their less active counterparts [1, 2]. Despite this, physical activity levels are known to decrease through childhood into adolescence [3, 4] and adulthood [5], with parents of young children known to be particularly inactive [6], especially compared to their childless counterparts [7, 8].

Parental activity has frequently been investigated as a correlate and determinant of child’s activity [9–11], given genetic [12], behavioural and social influences [9] parents have on their child’s behaviour [11]. Yet evidence of an association between parent and child physical activity is mixed [9–11], likely due to the widespread use of self-reported measures of physical activity. Studies assessing the link between objectively measured physical activity in parents and children are more scarce. Several smaller studies [13–15], and two larger studies conducted in UK [16] and US [17] preschool-aged children (3–5-year-olds) and their parents, suggest that there is a significant positive relationship between objectively measured activity levels in mother-child [13–17] and father-child pairs [13, 14, 17]. Similar results were also seen in older school-aged children (i.e. 5–11 year olds) in studies conducted using accelerometers [18, 19] and pedometers [20].

Very little information also exists about how the association between parent-child physical activity changes over time. This is particularly true of the important period when children transition from preschool into primary school, where children’s daily routine changes, they develop greater independence, and potentially engage in more organised activity. Only one aforementioned UK study has followed up 5–6-year-olds and their parents 3 years later [21], indicating that parental sedentary time/ MVPA at baseline did not predict later child behaviour (at 8–9 years), and change in parent MVPA or sedentary time was not associated with change in child activity behaviour [21]. Given the consistent cross-sectional association between parent-child activity levels, and that both children’s and parents’ physical activity levels decline over time [3–5], it is important to better establish how family members’ activity levels are associated as children age. Such information would be useful to encourage higher activity levels amongst families in early childhood, and prevent the subsequent decline known to occur in children as they age.

Using data from the UK Southampton Women’s Survey, this paper investigates the cross-sectional association between objectively measured maternal and 6-year-old children’s activity levels and assesses how this association differs by demographic and temporal factors. In addition, using a smaller prospective sample, it provides novel insight into how this association changed during the transition to primary school (from age 4–6).

## Methods

### Population

The Southampton Women’s Survey (SWS) is a population-based prospective cohort study located in Southampton, UK. Participant recruitment and data collection procedures are reported elsewhere [22]. At age 4 (March 2006–June 2009), a sub-study was conducted to investigate physical activity, with all SWS children who were aged 4 years during this period and their mothers (*n* = 730) invited to participate [16]. SWS children born after January 2000 were subsequently approached for an age-six visit (March 2007 – August 2012), where mothers and children were both asked to wear an activity monitor, and mothers completed a questionnaire assessing physical activity correlates. Ethical approval for SWS data collection at age 4 and 6 was granted by the Southampton and South West Hampshire Local Research Ethics Committee.

### Measures

As per the protocol for data collection at the age 4 physical activity sub-study [16], at the age-six visit, children (*n* = 802) and their mothers (*n* = 608) were fitted with a combined heart rate and movement sensor (Actiheart, Cambridge Neurotechnology Ltd., UK) to measure free-living physical activity. The monitor was secured to the chest and, for data storage reasons, set to record at 60-s epochs. Previously validated in children [23] and adults [24], participants were asked to wear the monitor continuously for 7 days, including during sleep and water-based activities. Monitors were returned by secure post.

### Outcome variables

Only accelerometer data were used for these analyses, as non-individually calibrated heart-rate data have been shown to explain little additional variation in estimates of free-living activity behaviour in young children [23]. Actiheart data were downloaded and processed using STATA 13/SE [25]. At both time points and for all participants, data periods of 100 min or more with zero-activity counts were removed [26], as were days with < 600 min of recording, with 10 h of activity being the cut-off to define a valid day [27]. All recordings between 11 pm and 6 am were removed, with those between 9 pm and 11 pm removed if they included more than 45 min of sedentary time, deemed to reflect the hours children spent sleeping. This method represents a conservative estimate of sleep time [28], whilst minimising an over-estimation of children’s sedentary time in the evenings. To assess the association between concurrently measured activity in children and mothers, data were matched exactly for hour and day of recording. All hours removed as sleep for children were also removed for their mother, ensuring direct comparison of activity levels.

Physical activity was described as time spent (min/day) in three broad intensity categories, using previously validated cutpoints to classify children’s activity level as sedentary (SED: < 20 counts per minute), light (LPA: > 20–460), and moderate-to-vigorous (MVPA: > 460) [29]. At the time of measurement, physical activity guidelines for children at age 4 were 180 min of any activity, and at age 6 were 60 min of MVPA per day respectively [30]. Therefore only SED and MVPA were used as outcomes in cross-sectional analyses, with SED, LPA and MVPA used in longitudinal analyses. Women’s activity was classified as SED: < 20 counts per minute; LPA: > 20–400; MVPA: > 400 [16, 31]. Both sets of cutpoints were scaled using a conversion factor of 5 from validation work using the Actigraph accelerometer (Actigraph, Pensacola, FL, USA) [32], with this scaling used previously in women and children of the same age [16].

### Moderating variables

Following appraisal of the evidence of activity in children [33, 34], and their mothers [6, 7, 35], a range of putative moderating variables were considered. Hour, time of the day/ week and season were obtained from the accelerometer output. Each day was split into three periods: morning (7-9 am), school (9-3 pm) and evening (3-11 pm). Season was defined as: winter: December–February; spring: March–May; summer: June–August; autumn: September–November. Child’s age, gender, and child’s and mother’s measured height and weight were recorded during the age 4 and age 6 visits [36]. For each time point, the latter were used to calculate mother’s and child’s body mass index (BMI) (kg/m^2^) and child’s BMI z-score [37]. For descriptive purposes participants were categorized as under-weight, normal or over-weight/obese using the International Obesity Task Force [38] and WHO [39] classifications for children and mothers, respectively.

Data from the maternal self-report questionnaire at age 4 were used to derive age mother left full-time education, classified as: < 16 years; 17–18 years and > 18 years. Data collected at age 4 and 6 were used to determine the presence of older or younger children living in the home at each time point: cohort child only; younger siblings; older siblings; older and younger siblings.

### Statistical analysis

Analyses were carried out using STATA/SE 14 [40]. Children and mothers with one or more shared valid day of activity data were included in analyses. Descriptive characteristics of the mother-child dyads at age 6 were calculated and compared a) against the original SWS cohort and b) for those providing longitudinal data at age 4 and 6. Sensitivity analyses were also conducted to compare whether dyads with > 1 or > 3 days of valid activity data differed. A significance level of 0.05 was set a priori for all tests.

#### Cross-sectional associations

Using children’s daily activity as the outcome, two-level random intercept models were used to model the association between children’s daily activity and maternal activity at the same intensity. Hierarchical models allow for variation across days (level 1) within mother-child pair (level 2) [41]. Correlations between observations were accounted for by allowing the intercept to vary randomly between children (i.e. level 2). Models were adjusted for child’s sex, z-BMI score and siblings in the home at age 6; age mother left full-time education; time of the week (weekday vs. weekend); and season. All covariates apart from z-BMI score were entered separately as an interaction term with maternal activity. In separate models, the association between maternal and child activity segmented across the day was then assessed for weekdays only. Limiting data to morning, school-time and evening segments, data were analysed in the same way as daily models (one record per segment per day (level 1) for each mother-child pair (level 2)).

#### Longitudinal associations

In the subset for whom physical activity was available at age 4 and 6 (*n* = 170), the association between maternal-child activity over the 2 year period was explored. For these analyses, at each time point, child’s activity levels were averaged over the measurement week, with an interaction term (age 4 or age 6) included to assess how the relationship between maternal-child physical activity differed by age. Models were adjusted for child’s sex, z-BMI score and siblings in the home at age 4, age mother left full-time education, time of week and season. The association between maternal and child activity over the 2 year period, segmented across the day (morning, school and evening) was also assessed, but due to the small sample size, interactions with demographic factors were not considered. Since season of assessment differed between baseline and follow-up for most (75%) of the mother-child dyads, as a sensitivity analysis, activity levels were residualised, standardising them for season. Only small differences were observed in regression coefficients (i.e. < 0.2), and we therefore present results from the original analyses for ease of interpretation.

For both cross-sectional and longitudinal analyses, sensitivity analyses were conducted to assess a) whether including only mother-child pairs with > 3 (vs. > 1) days of data and b) excluding data collected in August (UK school summer holidays) influenced our findings.

## Results

Of the 802 children and 608 women who returned activity monitors when the child was aged 6, 530 mother-child pairs provided valid activity data for one or more shared days (mean = 5.28 (SD:1.9) days; 13.6 (0.6) valid hours per day). Table 1 gives the characteristics of the mothers and children providing data at age 6 only, and at both age 4 and 6. Daily activity levels for the mothers and children at age 6 and change between age 4 and 6 are shown in Table 2, with activity levels segmented across the day at age 6 given in Table 3. Activity levels observed in children (and therefore mothers) did not differ at age 6 (or age 4 [16]) between those with > 1 and > 3 days of measurement; all pairs with > 1 shared day(s) of valid activity data were included in analyses.

**Table 1:** Descriptive characteristics for children and their mothers (*n* = 530)

	Cross-sectional (*n* = 530)		Prospective (*n* = 170)	
	Children^a^	Mothers^b^	Children	Mothers
Female (n (%))	257 (48.5)		85 (50)	
Age (years)	6.7 (0.3)	37.2 (3.6)	6.7 (0.3)	37.7 (3.7)
BMI (kg/m^2^)	16.1 (1.7)	24.4 (9.2)	16.2 (1.7)	26.7 (5.2)
Weight Status (n (%))				
Underweight	30 (5.7)		7 (4.2)	
Normal weight	408 (77.9)	264 (50.6)	130 (78.3)	56 (42.4)
Overweight/ obese	86 (16.4)	257 (49.3)	29 (17.5)	78 (57.5)
BMI z-score	0.25 (1.0)		0.32 (1.0)	
Age mother left full-time education (n (%))				
< 16 years		191 (36.1)		55 (32.4)
17–18 years		196 (37.1)		65 (38.2)
> 18 years		142 (26.8)		50 (29.4)
Other children in the home (n (%))				
None	218 (41.4)		71 (41.8)	
Older only	138 (26.0)		40 (23.5)	
Younger only	135 (25.5)		46 (27.1)	
Older and younger	39 (7.4)		13 (7.7)	

All values are mean (sd) unless stated otherwise; *sd* standard deviation, *a* Inclusive of 170 children in prospective cohort, *BMI* Body Mass Index; ^a^ Children included in the longitudinal analyses (compared with those providing valid activity data at age 6 only) were more likely to have a higher BMI at age 4 (16.8 vs. 16.1, *p* = 0.003) and be older at age 6 (6.7 vs. 6.6 years, *p* = 0.001); ^b^ Mothers providing data at both time points (vs. aged 6 only) were more likely to have a higher BMI (26.7 vs. 24.4 kg/m2, *p* = 0.03) and have left school later (29.4% vs. 26.8% leaving school after 18 years *p* = 0.04)

**Table 2:** Average daily activity levels for children and their mothers at age 6, and change between age 4–6

	Age 6		Change (Age 4–6)	
	Children	Mothers	Children	Mothers
Valid days	5.3 (1.9)		5.4 (1.8)	
Valid hours per day	13.6 (0.6)		13.6 (0.6)	
Sedentary time (minutes/day)	307.8 (3.2)^a^	426.2 (4.1)	42.1 (91.6)	4.5 (105.3)
Light physical activity (minutes/day)	445.3 (3.1)^b^	426.2 (4.1)	−57.0 (88.9)	−22.5 (98.8)
Moderate-to-vigorous physical activity (minutes/day)	65.4 (1.2)^c^	24.3 (0.83)	3.8 (56.2)	6.9 (15.8)
Percent of days activity guidelines met^d^	50.1	30.3	–	–
Percent of participants meeting PA guidelines on 50% of measurement days	57.5	3.2	–	–

All values are mean (sd) unless stated otherwise; sd: standard deviation; ^a^ difference by sex *p* = 0.132; ^b^ difference by sex *p* = 0.045; ^c^ difference by sex *p* < 0.001; ^d^ Children’s guideline: 60 min of MVPA, Adult: 30 min MVPA

**Table 3:** Average activity levels for children and their mothers at age 6, stratified by time of day and week

	Sedentary time (minutes)	Difference by sex	Moderate to vigorous physical activity (minutes)	Difference by sex
Morning (6-9 am)				
Children	60.3 (26.4)	*p* = 0.350	5.2 (6.1)	*p* = 0.043
Mothers	65.8 (30.2)		2.0 (3.5)	
School (9 am-3 pm)				
Children	109.7 (48.2)	*p* = 0.0006	34.7 (22.3)	*p* < 0.0001
Mothers	175.8 (64.9)		6.4 (8.4)	
Evening (3-11 pm)				
Children	135.5 (59.1)	*p* = 0.357	27.2 (22.7)	*p* < 0.0001
Mothers	184.9 (60.7)		4.8 (6.4)	
Weekday (6 am – 11 pm)				
Children	300.7 (93.7)	*p* = 0.764	69.2 (35.1)	*p* < 0.0001
Mothers	416.8 (114.0)		14.3 (13.5)	
Weekday (6 am – 11 pm)				
Children	321.0 (102.9)	*p* = 0.523	56.2 (42.3)	*p* < 0.0001
Mothers	449.7 (120.4)		9.3 (12.4)	

All values are mean (sd) unless stated otherwise; *sd* standard deviation

### Cross-sectional associations between child and maternal physical activity at age 6

At age 6, there was a direct positive association between mother-child activity levels indicating that for each extra minute of maternal activity, children engaged in 0.23 min more sedentary time and 0.53 min more MVPA (Table 4). There was a differential effect by time of the week (i.e. significant interaction effect), such that the association was stronger at the weekend (vs. weekdays: difference in effect SED: β_i_ = 0.07 [0.02, 0.12] minutes/day; MVPA: 0.44 [0.24, 0.64] minutes/day). For SED, there was a stronger association between in those children with older siblings (vs. none); for MVPA, the relationship was stronger for those who had both younger and older siblings (vs. none). A MVPA by season interaction indicated a stronger association in winter (vs. spring − 0.48 [− 0.82,-0.14]).

**Table 4:** Associations between child and maternal physical activity levels, and influence of temporal and demographic factors

	Sedentary time		Moderate to vigorous physical activity	
	β [95% C.I.] (minutes per day)	*p*-Value	β [95% C.I.] (minutes per day)	*p*-Value
Average daily activity^a^	0.23 [0.20,0.26]	< 0.001	0.53 [0.43,0.64]	< 0.001
Interaction terms				
Sex	−0.03 [− 0.09,0.03]	0.276	− 0.30 [− 0.50,-0.09]	0.004
Age mother left full-time education (ref < 16 years)				
17–18 years	0.06 [−0.01,0.13]	0.118	−0.07 [− 0.30,0.17]	0.583
> 18 years	− 0.02 [− 0.10,0.06]	0.652	−0.01 [− 0.27,0.24]	0.920
Siblings (ref: none)				
Younger only	−0.02 [− 0.10,0.06]	0.581	0.24 [− 0.04,0.52]	0.089
Older only	0.09 [0.01,0.16]	0.025	0.12 [−0.13,0.36]	0.346
Older and younger	0.09 [−0.04,0.22]	0.171	0.53 [0.12,0.94]	0.012
Time of the week (ref: weekday)	0.07 [0.02,0.12]	0.009	0.44 [0.24,0.64]	< 0.001
Season (ref: winter)				
Spring	−0.01 [−0.10,0.08]	0.963	−0.48 [− 0.82,-0.14]	0.005
Summer	0.03 [−0.06,0.12]	0.715	−0.01 [− 0.35,0.34]	0.965
Autumn	0.03 [−0.05,0.12]	0.784	−0.13 [− 0.46,0.20]	0.444
Association by time of day^b^				
Morning (6 am–9 am)	0.44 [0.40,0.49]	< 0.001	0.15[0.13,0.16]	< 0.001
School (9 am-3 pm)	0.06 [0.03,0.10]	< 0.001	0.02 [0.01,0.03]	< 0.001
Evening (3-11 pm)	0.40 [0.35,0.44]	< 0.001	0.06 [0.04,0.07]	< 0.001

*β* beta regression coefficient, *95% C.I.* 95% confidence interval; ^a^ models adjusted for child sex, weight status, age mother left full-time education, siblings at home, time of the week and season; ^b^ weekdays only, models adjusted for child sex, weight status, age mother left full-time education, siblings at home, and season

When analyses were stratified by time of day (Table 4), the association between maternal-child activity was weaker at all intensities during the school day (compared with morning and evenings). The association was strongest in the morning (SED: β_i_ = 0.44 [0.40, 0.49]; MVPA: 0.15[0.13, 0.16]).

### Longitudinal associations between mother-child activity between age 4 and 6

Table 5 shows that the association between mother and child activity overall did not differ by age for SED and LPA, but was weaker for MVPA at age 6 compared with age 4 (i.e. interaction with age: MVPA: − 0.16 [− 0.31, − 0.00]). Stratified analyses by time of day were limited to weekdays due to significant age by maternal interaction for weekdays only. This showed that overall change in the association between maternal-child MVPA appeared to be driven by a weaker relationship in the mornings (6-9 am) and during the school day at age 6 (9-3 pm). For SED and LPA the relationship was weaker in the evenings and in the mornings respectively at age 6.

**Table 5:** Prospective associations between child and maternal physical activity levels between age 4 and 6 years

	Sedentary time	*p*-Value	Light Physical Activity		Moderate to vigorous physical activity	*p*-Value
			β [95% C.I.] (minutes/day)	*p*-Value		
Daily^a^						
Maternal activity	0.23	< 0.001	0.21	< 0.001	0.45	< 0.001
	[0.18,0.28]		[0.16,0.26]		[0.31,0.58]	
Age 6 vs Age 4	57.30	< 0.001	−42.23	< 0.001	7.75	< 0.001
	[27.56,87.05]		[−67.12,-17.34]		[3.71,11.78]	
Interaction	−0.05	0.730	−0.02	0.258	**−0.16**	**0.042**
	[−0.12,0.02]		[−0.08,0.05]		**[−0.31,-0.00]**	
Morning^b^ (6-9 am)						
Maternal activity	0.41	< 0.001	0.39	< 0.001	0.17	< 0.001
	[0.35,0.48]		[0.33,0.45]		[0.14,0.19]	
Age 6 vs Age 4	9.33	0.001	1.71	0.513	0.44	< 0.001
	[3.87,14.78]		[−2.95,6.37]		[0.31,0.58]	
Interaction	−0.07	0.368	**−0.13**	0.003	**−0.07**	< 0.001
	[−0.16,0.01]		**[−0.21,-0.05]**		**[−0.10,-0.04]**	
School^b^ (9 am-3 pm)						
Maternal activity	0.04	0.038	0.04	0.080	0.04	< 0.001
	[0.01,0.09]		[−0.00,0.09]		[0.03,0.05]	
Age 6 vs Age 4	20.81	0.001	−25.41	< 0.001	0.78	0.021
	[9.89,31.72]		[−34.76,-16.06]		[0.55,1.02]	
Interaction	0.02	0.136	−0.03	0.637	**−0.03**	**0.010**
	[− 0.04,0.08]		[− 0.09,0.02]		**[− 0.04,-0.01]**	
Evening^b^ (3-11 pm)						
Maternal activity	0.20	< 0.001	0.14	< 0.001	0.04	< 0.001
	[0.13,0.26]		[0.08,0.20]		[0.02,0.05]	
Age 6 vs Age 4	24.69	0.001	−2.13	0.423	0.22	0.034
	[10.15,39.22]		[−13.74,9.48]		[0.01,0.42]	
Interaction	**−0.08**	0.035	−0.06	0.216	−0.01	0.076
	**[−0.16,-0.01]**		[−0.13,0.01]		[−0.03,0.00]	

*β* beta regression coefficient, *95% C.I.* 95% confidence interval; ^a^ Full day: > 10 h, model adjusted for child sex, weight status, age mother left full-time education, siblings at home, time of the week and season; ^b^ weekdays only, number of included hours indicated in brackets, all models adjusted for wear time, child sex, weight status, age mother left full-time education, siblings at home, and season; bold text indicates significant interaction effect for maternal physical activity and child age; data points: Morning: *n* = 1360; School: *n* = 1488; Evening *n* = 181

## Discussion

This is the first study to assess the association between mother-child activity levels during the transition to primary school. Cross-sectionally at age 6, there was a positive relationship between maternal-child activity, such that higher physical activity (and sedentary time) in mothers was associated with increases in the same behaviour in her child. For every 10 min of MVPA a mother did per day, her child did 5.2 min extra/day. This means that a child whose mother met her 30 min/day activity guideline accumulated 15 min MVPA extra/day compared to children of inactive mothers. Maternal-child associations were stronger at the weekend, and in the morning and evening compared to during school hours on weekdays, as might be expected as children are in school full-time at age 6. Longitudinally, the association between mother-child SED and LPA did not change between age 4 and 6, but it weakened for MVPA (i.e. was stronger at age 4) particularly during the school day.

As found previously in another large sample of UK 5–6-year-old children [42], and in this sample of children at age 4 [16], there was a positive association between mother-child sedentary behaviour and physical activity at age 6. The pattern of the relationship at age 6 differed from that seen at age 4 [16], with stronger associations at weekends (vs. weekdays); on weekdays, the association was strongest in the mornings and evenings. This indicates that when not in formal schooling, mothers and children influence each other’s activity. Although weaker, a positive association still remained during school time. This may be the result of factors such as mutual active travel together to and from school, but it also may indicate that the effect goes beyond joint activity and that active mothers are more likely to have generally more active children. Further research is needed to determine what drives this in the absence of possible co-participation to inform intervention development.

Previous research indicates differing associations between Australian mother and child activity levels by sex [43], with a UK study suggesting a stronger association between mother-daughter activity levels vs. mother-sons [42]. Here, we found that the relationship was stronger in mother-son pairs for MVPA, although an association for girls also existed. Having older siblings in the home was associated with a stronger relationship between mother-child SED, while having older and younger siblings was associated with a stronger relationship between mother-child MVPA. At age 4 in this sample, having older siblings was associated with greater MVPA in cohort children [44], and mothers’ LPA was positively associated with having children aged 4 and younger in the home [16]. It is plausible that as children (and siblings) age, the types of physical activity that they engage in as a family differs. Initially, having an older siblings may encourage physical activity in younger children, but as children age, and if an older sibling becomes more sedentary, this may also result in more sedentary behaviour in younger children and parents. It is also feasible that with greater numbers of children (i.e. older and younger siblings) in the home, families are more active as a whole. The number and age of children in the home therefore appears to influence both maternal and children’s physical activity levels, and consequently the way in which families engage in physical activity together.

Boosting physical activity in one of the mother-child pair may result in increased physical activity in the other, whilst feasibly having a positive influence on other members of the family (e.g. siblings, fathers). Family Systems Theory supports this, suggesting that an individual cannot be thought of in isolation, but rather as part of the family unit [45]. Behaviour of an individual is intrinsically linked to that of other family members, with positive physical activity behaviour in one person leading to others subsequently engaging in that behaviour themselves [45]. It should be noted that fathers (and also siblings) were not measured in our study, but have been shown to have a significant influence over children’s [8, 13, 42], and spouses’ [35], physical activity levels. Further high quality evidence is therefore needed to determine how fathers impact physical activity within families.

Longitudinally, we identified a weakening of the association between mother-child MVPA during the transition to school. This likely reflects the change in the types of activities that mother-child pairs engage in before and after children start formal schooling. Children may engage in higher intensity physical activity at school as indicated by increases in children’s MVPA overall at age 6 vs. age 4, whereas overall mothers engage in relatively little MVPA, fitting with our findings that the relationship was weaker during school hours. Similarly, we identified a weaker relationship between mother-child SED on weekday evenings. This may reflect an increase in children participating in (after-school) clubs, which parents have to transport their children to/ sit and watch, or parents engage in more sedentary activities in the evening (e.g. watching TV) as their children age.

Although UK guidelines at the time of measurement recommended children under 5 engage in 180 min of any activity, revised guidelines in a number of countries now advocate that children under 5 engage in 60 min of MVPA as part of this [46, 47]. Given our findings and that higher intensity activity is recommended for younger and older children and adults alike, encouraging activity of at least moderate intensity activity within families would allow all members to work towards meeting their daily activity guidelines. Moreover, greater consideration of how transitions, such as the start of formal schooling, influence where, when and how families are physically active together is now required to help with physical activity promotion in these populations.

### Strengths and limitations

Strengths of this study include the large population-based sample of over 500 participants, the inclusion of longitudinal data, and the use of objectively measured physical activity matched concurrently hour-for-hour. Twenty-four hour monitoring resulted in greater wear time than is common in this population (with previous studies typically removing monitors for water-based activities and sleep). The use of 60-s epochs, necessary to allow sufficient memory to record for 7 days when data were collected, may underestimate time spent in higher intensity activity in younger children [48], but is mitigated to some extent by combining intensities together to derive MVPA as was done here [49].

Participants were drawn from all socio-economic strata in Southampton and the surrounding areas, but as is common in cohort studies, mothers included in these analyses left formal education later and were older when they gave birth compared to the initial cohort. Due to the nature of data collection in the SWS cohort, sampling at age 4 and 6 differed. This resulted in a longitudinal subsample which was smaller than either cross-sectional cohorts, with differences among those providing data at age 6 only vs. at age 4 and 6 as noted in the results. In general, this does not appear to impact on the conclusions we can draw from these analyses, though the analyses may have been underpowered to detect interactions that might have been evident in a larger sample size. Overall, fewer children were overweight or obese here compared with the national average [50], and participants were predominately white British in line with the Southampton region (~ 82%) [51]. Although this does not introduce bias into the associations we identified within the dataset, caution is required when generalizing these findings to other populations.

## Conclusions

This study found a positive association between mother-child activity levels at age 6, which differed by time of the week, and age and number of siblings in the home. The relationship for MVPA weakened from age 4 to 6, possibly reflecting the differing activities children and mothers engage in during school and out-of-school hours, and increased independence as children grow older. Use of differing intervention strategies to promote family-based physical activity before and after the transition to formal schooling may therefore be warranted, given out-of-school physical activity, particularly in afternoon/ evenings constitutes a substantial component of children’s (and mothers’) overall physical activity.

## Abbreviations

BMI: Body mass index
CI: Confidence interval
LPA: Light physical activity
MVPA: Moderate to vigorous physical activity
SED: Sedentary
SWS: Southampton Women’s Survey
